# Amyloid goiter - A rare case report and literature review

**DOI:** 10.1016/j.amsu.2020.08.004

**Published:** 2020-08-13

**Authors:** Eisa Lari, Waleed Burhamah, Ali Lari, Salman Alsafran, Ali Ismail

**Affiliations:** General Surgery Department, Jaber Al-Ahmad Hospital, Ministry of Health Kuwati, PO: 091710, Kuwait

**Keywords:** Amyloid, Goiter, Amyloidosis, Thyroid, Thyroidectomy

## Abstract

**Introduction:**

Amyloid goiter is a rare presentation of thyroid swelling, which occurs with either primary or secondary amyloidosis. This condition must be differentiated from other types of goiters or malignancies. Even though the thyroid is extensively involved by amyloid, patients are usually euthyroid, but many different presentations and overlaps have been reported. Currently the treatment is surgical resection of the thyroid gland

**Case presentation:**

We report a case of a 53-year-old previously healthy male who presented with a 2 year history of a progressively enlarging painless neck swelling. The patient was euthyroid and denied any associated symptoms. The patient subsequently underwent an uneventful total thyroidectomy along with an unremarkable follow up and was diagnosed with primary amyloidosis involving only the thyroid gland confirmed by histopathology.

**Discussion and conclusion:**

Amyloid goiter is a rare entity; a high index of suspicion is required in patients with an enlarging thyroid gland and a concomitant history of chronic inflammatory processes or plasma cell dyscrasia. FNA biopsy should be performed to exclude the top differential of primary thyroid malignancy. Thyroidectomy is necessary for definitive diagnosis and symptom relief. Every effort should be made to delineate the extent of the disease, and in those previously healthy plasma cell dyscrasia should be excluded

## Highlights

• We present a rare case of amyloid goiter, detailing our work up, results and management.
• The majority of patients are euthyroid at presentation.
• Thyroid malignancy must be ruled out as a differential.
• Thyroidectomy is necessary to reach a definitive diagnosis and/or for symptom relief.
• Systemic workup required; delineating extent of disease.

## 1 Introduction

Amyloidosis is defined as the accumulation of amorphous, proteinaceous material in different parts of the body. Amyloid can be deposited in the thyroid gland in such quantities to cause a clinically apparent enlargement of the gland known as Amyloid goiter [1]. This rare entity was first described in 1858 by Beckman, later Eiselberg in 1904 introduced the term ‘Amyloid Goiter’ [2]. Deposition of Amyloid in the thyroid gland occurs in 15% of primary amyloidosis and 20% in secondary amyloidosis [3].

Even though involvement of the thyroid gland by amyloid is quite common, clinically significant enlargement of the thyroid due to amyloid deposition is extremely rare, and most cases are not diagnosed prior to surgery [4].

Definite histopathological diagnosis is generally only possible postoperatively, but a high index of suspicion should be maintained whenever amyloid deposits are seen on needle aspiration and cytology.

Aim: We present an extremely rare case of a euthyroid patient with primary (AL amyloidosis) involving only the thyroid gland with no systemic involvement resulting in an amyloid goiter. We detail the importance of a detailed workup in order to exclude an underlying disease and/or delineate the extent of the disease.

This case report was reported in line with the SCARE criteria [36].

## 2 Case Presentation

A 53-year-old, previously healthy gentleman, presented to the general surgery out-patient department complaining of a 2-year history of a painless neck swelling which has been increasing in size, with no symptoms of obstruction or hypo/hyperthyroidism. Review of systems revealed no other symptoms.

On physical examination a swelling was noted in the anterior aspect of the neck. Palpation revealed a bilateral, non-tender, firm, multi-nodular swelling. No skin changes, bruit or retrosternal extension was present. Systemic physical examination was unremarkable.

Laboratory results showed normal Thyroid function test, complete Blood Count, serum electrolytes and Renal function Test, and negative Thyroid Specific anti-bodies.

Neck ultrasound revealed an enlarged thyroid gland with hyperechogenicity of the parenchyma. The size of the left lobe was 104 mm × 33 mm x 50 mm and right was 89 mm × 65 mm x 43 mm.

A computerized tomography (CT) scan of the neck and chest showed diffuse enlargement of the thyroid gland and a heterogeneous enhancement in both thyroid lobes. The thyroid parenchyma appeared to be replaced near-totally by fatty tissue. No mediastinal extension of the thyroid gland and no lymphadenopathy appreciated.

A fine-needle aspiration cytology (FNAC) of the thyroid revealed numerous fragments of adipose tissue, with clusters of benign looking epithelial cells, with some in follicular pattern resembling thyroid follicles.

The patient subsequently underwent a total thyroidectomy by a senior endocrine surgeon. We ensured preservation of the parathyroid glands, which were normal in size, and the recurrent laryngeal nerves on both sides. Intra-operative findings were significant for an enlarged nodular thyroid gland with diffuse lipomatous change. The post-operative course was uneventful, and the patient was discharge home on post-operative day 4.

**Fig. 1:**
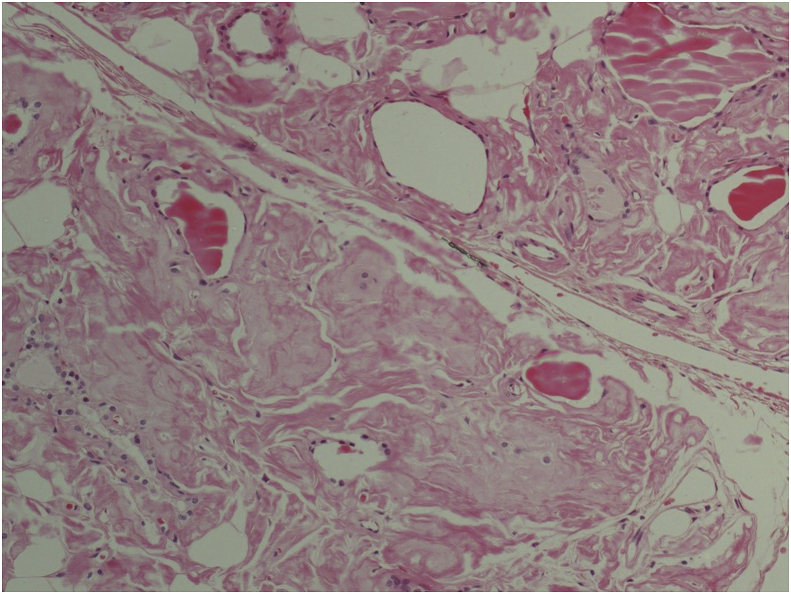
Haematoxylin and Eosin stained section showing deposition of acellular amorphous eosinophilic material (Amyloid).

**Fig. 2:**
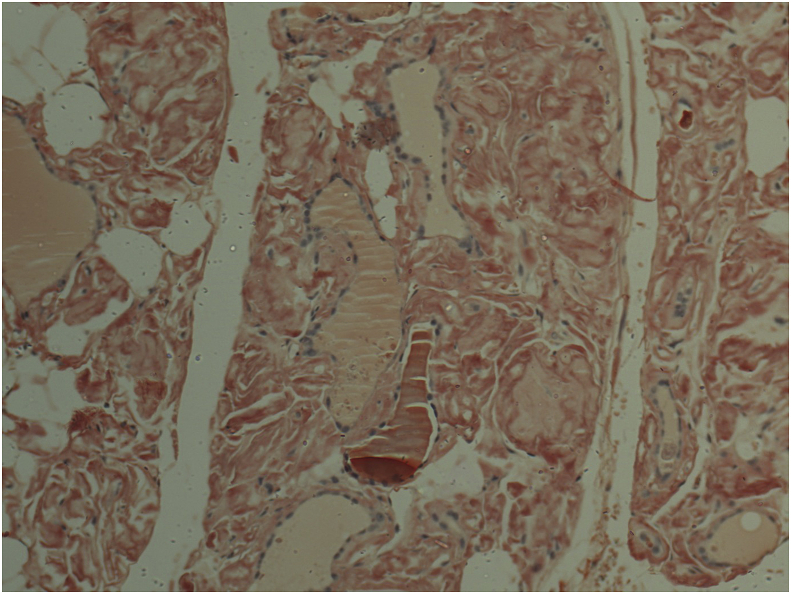
Congo Red staining renders amyloid deposits salmon pink. (For interpretation of the references to colour in this figure legend, the reader is referred to the Web version of this article.)

**Fig. 3:**
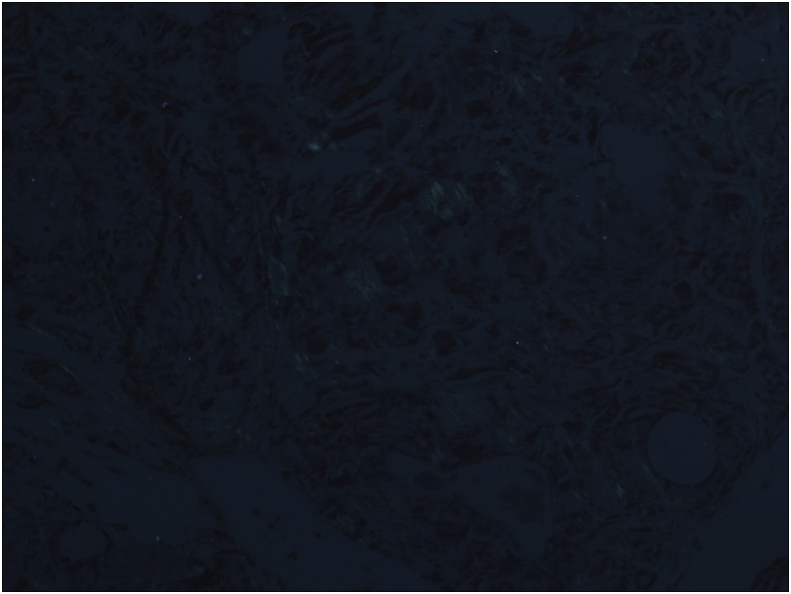
Apple-green birefringence under polarized microscopy. (For interpretation of the references to colour in this figure legend, the reader is referred to the Web version of this article.)

Grossly the specimen showed the right lobe measuring 10x5x5cm, the left lobe measuring 11 × 5 × 5cm. The thyroid surface was nodular and soft in consistency. Histopathology report revealed diffuse infiltration by mature adipose tissue, with attenuated scattered thyroid follicles. An extracellular eosinophilic amorphous substance deposited in the hyalinized stroma was noted. The extracellular substance proved to be positive by the Congo red stain and green birefringence under polarized light microscopy. (Fig. 1, Fig. 2, Fig. 3).

Immunohistochemistry revealed the AL type amyloidosis, thus, a diagnosis of amyloid goiter was made.

The patient was further investigated for the extent of amyloidosis and possible underlying diseases. An ultrasound of the abdomen, urinalysis, and a skeletal survey were all normal. A serum immune-electrophoresis revealed no monoclonal gammopathy.

A pre operative chest X-ray and electrocardiogram were normal. The pre operative CT chest showed no abnormalities of the heart. Patient has undergone an echocardiogram 3 months prior to surgery, showing no dilation, hypertrophy or diastolic dysfunctions.

Hence a final diagnosis of primary amyloidosis (AL) involving only the thyroid gland was made. Follow up in out-patient clinic 2 weeks later was unremarkable and patient was satisfied with no current concerns.

## 3 Discussion

Amyloidosis is a group of heterogeneous diseases resulting from the pathological deposition of an insoluble protein known as amyloid [5,6]. Amyloid is derived from soluble precursors that undergo a conformational change resulting in a protein of a beta pleated sheet configuration [[5], [6], [7]]. It deposits in the extra-cellular space of various organs, this compromises function and creates a mass effect. It is a diverse disease with many subtypes, each with a specific organ predilection. Therefore it can present with a wide spectrum of signs and symptoms.

‘Amyloid goiter’ is the deposition of amyloid in the glandular parenchyma of the thyroid gland causing a clinically apparent enlargement [1,8,9].

A typical histological picture will show extensive widespread infiltration [10,11] with evidence of follicular distortion and fatty metaplasia [10]. The exact mechanism responsible for fatty metaplasia is unknown. A theory suggests that amyloid deposition compromises capillary function resulting in relative ischemia; this triggers stromal fibroblasts to undergo metaplasia [12,13].

Although microscopic amyloid deposition in the thyroid gland is common in patients with certain conditions, amyloid deposition resulting in an overt goiter is a rare [6,12,14,15]. Evidence of thyroid micro deposits is seen in more than 80% of medullary thyroid cancers [16,17] and in 30–80% of patients with amyloidosis [18]. Those are typically incidental findings not leading to Amyloid Goiter [11].

The 2 main subtypes of amyloidosis implicated in amyloid goiter are primary (AL amyloidosis) and secondary amyloidosis (AA amyloidosis) [5,6].

Primary amyloidosis is linked to disorders of plasma cells collectively known as plasma cell dyscrasia [5,6]. Plasma cells play an integral part in our immune system, their function is to produce antibodies. Antibodies are proteins composed of 2 subunits, a light and a heavy chain, their function is to recognize and attack pathogens. In Plasma cell dyscrasia there is over production of plasma cells, antibodies and misshapen light chains. Light chains deposit in tissues in the form of AL amyloid [5,6]. The deposition can either be in a single organ (localized primary amyloidosis) or in multiple organs (systemic primary amyloidosis). Localized primary amyloidosis presenting with an isolated amyloid goiter is extremely rare [19,20]. This was seen in our patient, in which investigations failed to identify systemic involvement of AL amyloid.

Secondary amyloidosis is associated with chronic inflammatory conditions [5,6]. The acute phase reactant, serum amyloid A, is produced in excess and deposits in various organs in the form of amyloid A [5,6].

In developed countries the most common cause of amyloid goiter is primary amyloidosis [21,22], however in the developing world secondary amyloidosis is a more common cause. In secondary amyloidosis, amyloid goiter has been described to occur as a sequela of long standing Familial Mediterranean Fever [4,23], Inflammatory bowel disease [24], rheumatoid arthritis [24], TB [1] and bronchiectasis [25]. Overall amyloid goiter occurs more often in patients with primary compared to those with secondary amyloidosis [11].

Amyloid goiter is a rare entity and there is paucity in the literature detailing its clinical course and management. Patients will usually present with a firm, non-tender, rapidly growing swelling over weeks to years [5]. The swelling can be unilateral or bilateral, diffuse or nodular [5], and can be accompanied by dysphagia, dysphonia or dyspnea [6,11,20,26]. With such clinical course, malignancy, especially anaplastic thyroid cancer should be the topmost culprit differential diagnosis, and initial investigations should aim to rule out malignancy. However amyloid goiter should be on the list of differentials, especially in patients with longstanding inflammatory conditions or hematological malignancy. Most patients are euthyroid like in our case, however this is not the rule, as some patients may present with a hyperthyroid [28] or hypothyroid state [4,10,12,23,27,29].

Sonography, computerized tomography (CT) scans and magnetic resonance imaging scans (MRI) have been used to investigate goiters [30]. Imaging patterns in amyloid goiter may vary according to the proportion of adipose tissue relative to amyloid [30]. As seen with our patient, cases in which adipose tissue predominates, diffuse fat-equivalent hypo density with or without calcifications can be seen on CT scan [30]. On MRI an increased signal intensity will be seen on T1 and T2 weighted images [30]. In cases with amyloid deposition, complex or hypoechoic masses are seen on ultrasound [30]. Proteinaceous substances within the nodule can cause high intensity on T1-weighted images, and fibrillar amyloid structures can lead to increased intensity on T2-weighted images [30].

Fine needle aspirate cytology (FNA) is usually performed as part of the work up for thyroid nodules. In amyloid goiter, FNA cytology will show abundant irregular fragments of a pink amorphous material. When compared to colloid amyloid will appear more solid and hyaline-like [5]. It is estimated that 10–40% FNAs performed on amyloid goiters will only show atypical follicular cells [5] Unfortunately this may limit the ability of FNA to distinguish between amyloid goiter and medullary carcinoma [9,20]. Therefore, obtaining a precise diagnosis of amyloid goiter requires histopathology [20]. We encountered a similar situation in which FNAC was inadequate in leading to a diagnosis. The diagnosis of amyloid goiter is usually confirmed post operatively.

Histopathological samples will show eosinophilic amorphous deposits in the parafollicular areas. Those deposits will stain positively with Congo red dye and result in an apple-green birefringence when viewed under polarized light [5,6,19]. The presence of amyloid in a thyroid sample, along with its clinical course, should raise the suspicion of medullary cancer, which should be ruled out. Using a calcitonin immune-stain in medullary thyroid cancer amyloid will be limited to an intra tumoural distribution as opposed to a wide distribution in amyloid goiter and the absence of tumor cells [11]. Subsequently immunohistochemistry can then be used to differentiate between types of amyloid [28].

Once the diagnosis of amyloid goiter is established the extent of the disease has to be delineated [20]. Whether AL or AA amyloidosis is diagnosed, the patient should also be screened for predisposing conditions, as amyloid goiter can be the first presentation of a long-standing disease. [5,11,20.24,31] This was seen in a case by J. Orrego et al. [5]. His previously healthy patient presented with a thyroid swelling and was diagnosed with amyloid goiter AL type. Postoperative investigations revealed long standing monoclonal gammopathy of undetermined significance with no other systemic involvement of amyloid. K. Hill et al. [11] presented a case of amyloid goiter in which post-operative investigations revealed multiple myeloma.

Since our patient was previously healthy and was diagnosed with AL amyloidosis, we performed investigations to rule out associated hematological malignancies and investigate the extent of amyloidosis. Since all investigations were negative, our patient was given a diagnosis of primary amyloidosis localized to the thyroid gland.

In AL amyloidosis as a cause of amyloid goiter, three main pathological processes have been described [5]. First; Patients with primary thyroid lymphoma and plasmacytic differentiation [32]. The monoclonal, plasma cells produce light chains that deposit as amyloid [32]. In such cases amyloid deposits will be confined to the thyroid gland [32]. Second, patients with known or undiagnosed plasma cell dyscrasias, AL amyloid will deposit systemically and can manifest as amyloid goiter [5] or in rare cases deposition will be limited to the thyroid gland. This is similar to what was presented by J. Orrego et al. [5]. Third, a subset of patients with no identifiable cause of AL amyloidosis [19]. This is seen in our patient and cases presented by K.H Joung et al. and [20] C.Aparna et al. [31]. Immunohistochemistry revealed AL amyloidosis, further evaluation ruled out systemic organ involvement and the presence of plasma dyscrasia.

Typically a thyroidectomy is the treatment of choice in patients with an amyloid goiter if compressive symptoms are present, there are concerns of malignancy and considering the status of the current amyloid disease, if previously diagnosed. However, the definitive diagnosis usually requires a thorough histological evaluation of the thyroid [20]. In our patient, a thyroidectomy was performed in order to reach an accurate histological diagnosis and due to the cosmetic concern associated with the goiter.

It is of note to mention that in patients with amyloid goiter it is exceedingly rare to have clinically evident parathyroid enlargement due to amyloid deposition [11]. Four previous cases were described in the literature [11,[33], [34], [35]]. In all 4 cases the pre-operative laboratory investigations, including parathyroid hormone levels, were all normal. An enlarged parathyroid gland was only seen intra-operatively. This stresses the importance of examining the size of the parathyroid glands during surgery for amyloid goiter.

## 4 Conclusions

Amyloid goiter is a rare entity; a high index of suspicion is required in patients with an enlarging thyroid gland and a concomitant history of chronic inflammatory processes or plasma cell dyscrasia. FNA biopsy should be performed to exclude the top differential of primary thyroid malignancy. Definitive diagnosis typically occurs after thyroidectomy and histological analysis. Every effort should be made to delineate the extent of the disease, and in those previously healthy plasma cell dyscrasia should be excluded.

## Informed consent

Written informed consent was obtained from the patient for the publication of this case report and any accompanying images. A copy of the written consent is available for review by the Editor-in-Chief of this journal on request.

## Registration of research studies

Not applicable.

## Ethical approval

Ethical approval has been exempted by our institution, as this publication is a case report, provided that the patient's informed written consent is available.

## Author contribution

Eisa lari: case data collection, literature review.

Waleed Burhamah: literature review, writing the paper

Ali lari: writing the paper

Salmanan AlSafran/Ali ismail: Editing and supervision

## Provenance and peer review

Not commissioned, externally peer review.

## Financial disclosure

This research did not receive any specific grant from funding agencies in the public, commercial, or not-for-profit sectors.

## Declaration of competing interest

The authors declare no conflict of interest.
